# Prevalence and incidence of vector-borne pathogens in unprotected dogs in two Brazilian regions

**DOI:** 10.1186/s13071-020-04056-8

**Published:** 2020-04-21

**Authors:** Filipe Dantas-Torres, Luciana Aguiar Figueredo, Kamila Gaudêncio da Silva Sales, Débora Elienai de Oliveira Miranda, Joanna Lúcia de Almeida Alexandre, Yury Yzabella da Silva, Lidiane Gomes da Silva, Guilherme Ribeiro Valle, Vitor Márcio Ribeiro, Domenico Otranto, Katrin Deuster, Matthias Pollmeier, Gertraut Altreuther

**Affiliations:** 1 Department of Immunology, Aggeu Magalhães Institute, Oswaldo Cruz Foundation (Fiocruz), Recife, Brazil; 2 Centro Universitário do Vale do Ipojuca (UNIFAVIP/Wyden), Caruaru, Pernambuco Brazil; 3 Veterinary School, Pontifical Catholic University of Minas Gerais, Betim, Brazil; 4 Department of Veterinary Medicine, Università degli Studi di Bari, Valenzano, Bari, Italy; 5 Faculty of Veterinary Sciences, Bu-Ali Sina University, Hamedan, Iran; 6 Bayer Animal Health GmbH, Leverkusen, Germany

**Keywords:** *Anaplasma*, *Babesia*, *Dirofilaria*, *Ehrlichia*, *Leishmania*, Dogs, Prevalence, Incidence

## Abstract

**Background:**

Various vector-borne pathogens (VBPs) affect dogs worldwide, with their diversity and force of infection being usually higher in the tropics. Cross-sectional studies have been conducted to investigate the prevalence of VBPs in dogs, but data from longitudinal studies are scarce. Herein, we assessed the prevalence and the year-crude incidence (YCI) of *Leishmania* spp. and other VBPs in privately-owned dogs from two geographical regions of Brazil.

**Methods:**

A total of 823 dogs were initially screened for *Leishmania* spp. by both serology and polymerase chain reaction (PCR). From the negatives, 307 (103 from São Joaquim de Bicas, Minas Gerais, and 204 from Goiana, Pernambuco) were randomly selected for the longitudinal study. These dogs were tested for various VBPs at baseline, after 8 and 12 months.

**Results:**

Out of 823 dogs initially screened, 131 (15.9%) were positive for *Leishmania* spp. Out of the 307 dogs enrolled in the longitudinal study, 120 (39.1%) were lost for different reasons (e.g. animal death, owner decision, and lost to follow-up). In São Joaquim de Bicas, the baseline prevalence and YCI were as follows: 16.5% and 7.1% for *Anaplasma* spp.; 81.6% and 100% for *Babesia* spp.; 0% and 1.3% (only one faint positive) for *Dirofilaria immitis*; 37.9% and 22.9% for *Ehrlichia* spp.; 19.5% and 43.8% for *Leishmania* spp. In Goiana, the baseline prevalence and YCI were as follows: 45.1% and 38.3% for *Anaplasma* spp.; 79.9% and 96.0% for *Babesia* spp.; 36.3% and 39.8% for *D. immitis*; 64.7% and 58.5% for *Ehrlichia* spp.; 14.7% and 19.6% for *Leishmania* spp. Anti-*Borrelia burgdorferi* antibodies were not detected in any of the samples tested herein. The prevalence and YCI of *Anaplasma* spp., *D. immitis* and *Ehrlichia* spp. were significantly higher in Goiana. In contrast, the YCI of *Leishmania* spp. infection was significantly higher in São Joaquim de Bicas.

**Conclusions:**

We confirmed a high prevalence and YCI of various VBPs among privately-owned dogs in two geographical regions of Brazil. Our data also indicate that the risk of infection varies significantly for individual VBPs and between the regions, which may be related to several factors that are still poorly understood.
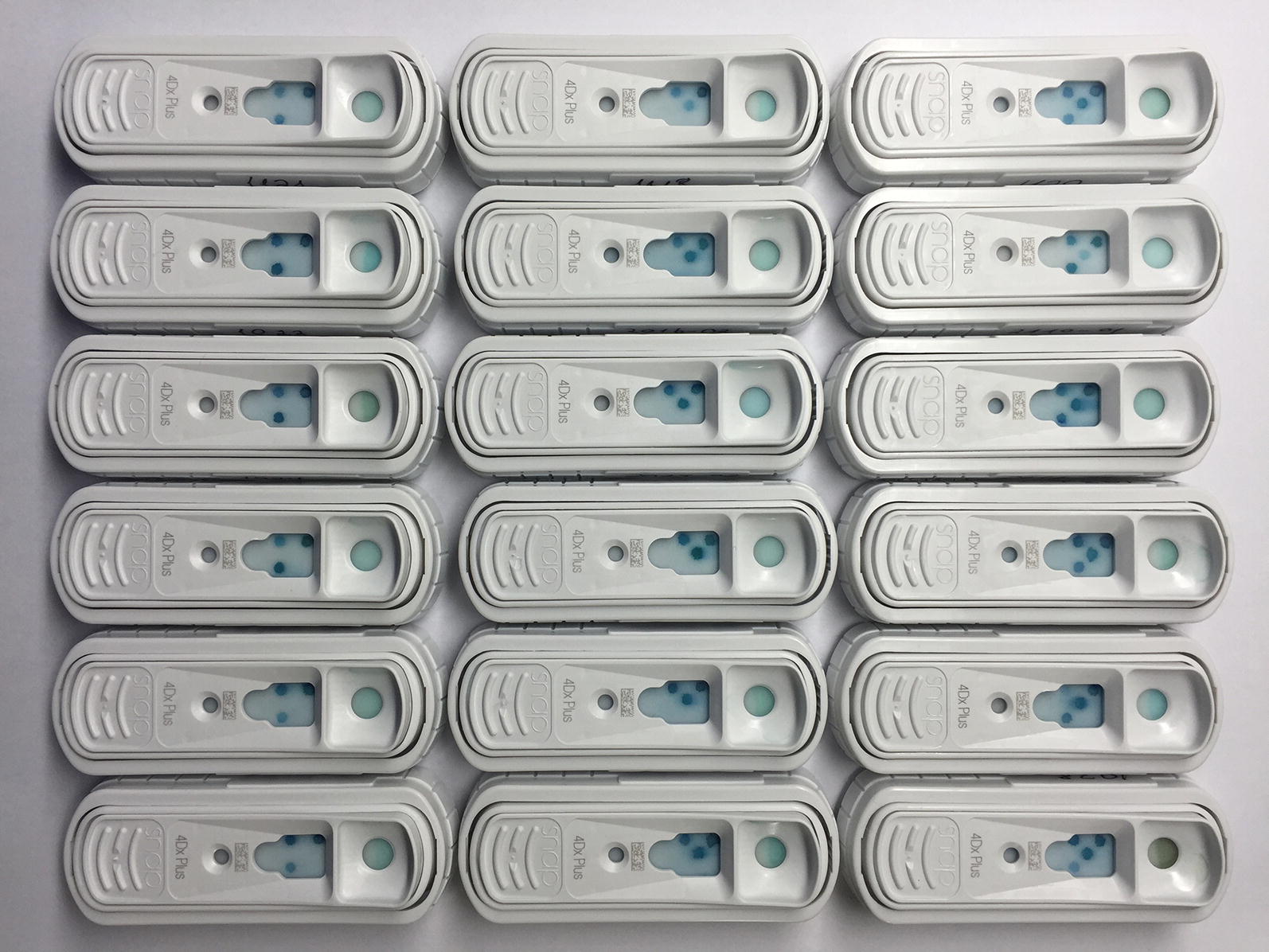

## Background

A wide range of vector-borne pathogens (VBPs), including bacteria, protozoa and filarial nematodes, can infect and eventually cause overt disease in domestic dogs worldwide [1–4]. These pathogens are transmitted to dogs, and eventually to other hosts, through various arthropod vectors, such as ticks, mosquitoes, phlebotomine sand flies, fleas, lice, and triatomine bugs [5].

Due to its unique climate and landscape types [6], the tropics are unique also in terms of diversity and abundance of arthropod vectors and their associated pathogens. For instance, an extraordinary diversity of ticks, mosquitoes, and phlebotomine sand flies may feed on dogs in the tropics, with many of these species restricted to this climate zone [7]. Moreover, the favourable climate found in most of the tropics provides the opportunity for VBP transmission to occur during the entire year [8], further increasing the risk of infection in dogs and, eventually, in humans [9, 10].

Brazil is an epicentre of VBP transmission in Latin America and Caribbean [11], not only in dogs, but also in humans [7]. Indeed, diseases like leishmaniasis, Chagas disease, malaria, dengue fever, and lymphatic filariasis, just to mention a few, are still responsible for a heavy burden, affecting disproportionally the poorest of the poor [12]. Dogs living in Brazil are also afflicted by numerous VBPs such as *Babesia vogeli*, *Dirofilaria immitis*, *Ehrlichia canis* and *Leishmania infantum* [7]. Additionally, they are also affected by pathogens that are restricted to Latin America, including *Leishmania amazonensis*, *Leishmania braziliensis* and *Rangelia vitalii* [7].

While several cross-sectional studies on VBPs infecting dogs have been conducted in the tropics, longitudinal studies are very scant and, for some pathogens, virtually inexistent. For instance, a few longitudinal studies on *L. infantum* infection in dogs have been conducted in Brazil (e.g. [13, 14]), a zoonotic parasite that still affects and kills thousands of Brazilians every year [15]. As a result, there is very limited information about the annual incidence of VBP infections in dogs, in spite of the large number of cross-sectional studies available in the literature (e.g. [16–20]). Prevalence data cannot *per se* be used to infer incidence, also considering that seroconversion may take months to occur and that antibodies produced against certain pathogens may last for months.

In this context, we estimated the year-crude incidence (YCI) of infection by *Leishmania* spp. in dogs from two municipalities of Brazil, based on data gathered from two cohorts of privately-owned dogs followed up for 1 year and whose new infections were diagnosed by serological and molecular tests. Additionally, infections by other VBPs were also investigated.

## Methods

### Study areas

This study was conducted from September 2015 to November 2016, in two urban areas. The first site was the municipality of Goiana (7°33′39″S, 35°0′10″W; altitude: 13 m), located ~ 62 km from Recife, the capital of Pernambuco State, north-eastern Brazil. Goiana has a tropical savanna climate with dry-summer characteristics, which corresponds to the Köppen climate classification categories “Aw” and “As”. The mean annual temperature and precipitation are 24.9 °C and 1924 mm, respectively. The mean monthly temperature ranges from 23.3 °C to 26 °C, whereas the mean monthly precipitation ranges from 46 mm to 307 mm.

The second site was the municipality of São Joaquim de Bicas (20°02′56″S, 44°16′26″W, altitude: 755 m), located ~ 45 km far from Belo Horizonte, the capital of Minas Gerais State, south-eastern Brazil. São Joaquim de Bicas has a humid subtropical climate with dry-winter characteristics, which corresponds to the Köppen climate classification category “Cwa”. The mean annual temperature and precipitation are 21.5 °C and 1348 mm, respectively. The mean monthly temperature ranges from 18.3 °C to 23.9 °C, whereas the mean monthly precipitation ranges from 10 mm to 287 mm.

These municipalities were chosen because a previous cross-sectional study confirmed the presence of various VBPs in privately-owned dogs [19]. Further details on these sites can be found elsewhere [19].

### Dog population and sampling

The study population included initially 823 privately-owned dogs, which were screened for anti-*Leishmania* spp. antibodies. Part (*n* = 632) of these dogs was also tested for *Leishmania* minicircle kinetoplast DNA (kDNA) by real-time PCR. From the negatives to both serology and PCR, and complying with some inclusion criteria (i.e. dogs should be in general good healthy, non-fractious, untreated with ectoparasiticides with known efficacy against VBPs, availability of written owner consent form), 307 (168 males and 139 females) dogs were randomly selected for the longitudinal study. Selected dogs were mostly mongrels, with age ranging from 2 months to 13 years (average = 2.7 years). Except for 76 dogs whose owners reported (at least once) the use of ectoparasiticides (39 in Goiana, 37 in São Joaquim de Bicas), all dogs were not treated to prevent ectoparasite infestations throughout the entire observation period.

At baseline, after 8 and 12 months, a 5 ml blood sample was collected from each dog, from the brachial, jugular or other suitable vein. Approximately 3 ml of blood were added to BD SST™ gel tubes and the remaining blood (2 ml) were collected in EDTA (purple cap) collection tubes. At the laboratory, BD SST™ gel tubes were centrifuged at 2000× *g* for 10 min for serum separation. Aliquots of sera were then immediately tested as described below. Remaining serum samples and EDTA-blood samples were frozen at − 20 °C.

### Serological testing

For the initial screening, dogs were tested for antibodies to *Leishmania* spp. using at least one of the following tests: ELISA/S7^®^ (Biogene), DPP LVC (Bio-Manguinhos), Alere Leishmaniose Ac Test (Alere), SNAP^®^ Leishmania Test (IDEXX Laboratories, Maine, USA). All dogs randomly selected for the longitudinal study had to be negative by the SNAP^®^ Leishmania Test (and also by PCR), which was the one used to retest the dogs after 8 and 12 months.

All dogs included in the longitudinal study were also tested (at baseline, after 8 and 12 months) by a rapid ELISA (SNAP^®^ 4Dx Plus Test, IDEXX Laboratories, Maine, USA) that detected antibodies to *Anaplasma* spp. (*A. platys*/*A. phagocytophilum*), *Ehrlichia* spp. (*E. canis*/*E. ewingii*), and *Borrelia burgdorferi*, and antigens of *Dirofilaria immitis*. Likewise, dogs were tested by an indirect immunofluorescence (IFA) that detected antibodies to *Babesia* spp. (*Babesia canis* IFA IgG Antibody Kit, Fuller Laboratories), with a cut-off of 1:50. All serological tests were performed using serum samples and according to the manufacturer’s instructions.

### Molecular testing

EDTA-treated blood samples collected from dogs were subjected to DNA extraction, using a commercial kit (PureLink^®^ Mini Kit, Invitrogen, Carlsbad, CA, USA), according to the manufacturer’s instructions. The quantity and purity of the extracted DNA were assessed using a NanoDrop 2000c Spectrophotometer (Thermo Fisher Scientific, Waltham, MA, USA) and samples were then stored at − 20 °C.

*Leishmania* spp. kDNA was detected by real-time PCR as described elsewhere [20, 21], using the primers LEISH-1 (5′-AAC TTT TCT GGT CCT CCG GGT AG-3′) and LEISH-2 (5′-ACC CCC AGT TTC CCG CC-3′) and the TaqMan^®^ probe FAM-5′-AAA AAT GGG TGC AGA AAT-3′-non-fluorescent quencher-MGB [21]. Each reaction contained a final volume of 15 μl, including 7.5 μl of TaqMan^®^ Fast Advanced Master Mix (Applied Biosystems, Carlsbad, CA, USA), 1.35 μl of each primer (at a concentration of 900 nM), 0.3 μl of probe (at 200 nM), 2 μl of DNA sample, 2.5 μl of DNA-free water. Thermal cycling conditions were as follows: denaturation at 95 °C for 20 s, and 40 cycles at 95 °C for 1 s and 60 °C for 20 s [22]. A standard curve was prepared from *L. infantum* genomic DNA (MHOM/BR/76/M4192) at different concentrations (1 ng, 100 pg, 10 pg, 1 pg, 100 fg, 10 fg, 1 fg, 0.1 fg and 0.01 fg per reaction). A master mix with no DNA was used as no template control (NTC). Real-time PCR reactions were performed using QuantStudio^®^ 5 Real-Time PCR system (Applied Biosystems, Foster City, CA, USA) and results were analysed using QuantStudio Design and Analysis Software v1.4 (Applied Biosystems, Foster City, CA, USA).

*Babesia* spp. DNA was detected by conventional PCR using the primers BcanisF (5′-GCA TTT AGC GAT GGA CCA TTC AAG-3′) and Bcommon-R (5′-CCT GTA TTG TTA TTT CTT GTC ACT ACC TC-3′), as described elsewhere [23]. Each reaction contained 8.5 μl of DNA-free water, 12.5 μl of GoTaq^®^ Colorless Master Mix (Promega, Madison, USA), 1.0 μl of each primer at a concentration of 25 pmol/μl and 2 μl of DNA sample. Thermal cycling conditions were as follows: initial denaturation at 95 °C for 2 min, followed by 35 cycles of 95 °C for 45 s, 60 °C for 45 s and 72 °C for 1 min, and a final extension step at 72 °C for 5 min. DNA extracted from the blood of a dog infected by *B. vogeli* was used as positive control and a master mix with no DNA as NTC.

*Anaplasma platys* DNA was detected by conventional PCR using the GroAplatys-35s (5′- AGC GTA GTC CGA TTC TCC AGT TTT-3′) and GroAplatys-550as (5′- TCG CCG TTA GCA GAG ATG GTA G-3′), as described elsewhere [24]. Each reaction contained 7.5 μl of DNA-free water, 12.5 μl of GoTaq^®^ Colorless Master Mix (Promega, Madison, USA), 1.5 μl of each primer at a concentration of 10 pmol/μl and 2 μl of DNA sample. Thermal cycling conditions were as follows: denaturation at 95 °C for 1 min, followed by 55 cycles of 94 °C for 15 s, 62 °C for 15 s and 72 °C for 15 s, and a final extension step at 72 °C for 7 min. DNA extracted from the blood of a dog infected by *A. platys* was used as positive control and a master mix with no DNA as NTC.

*Ehrlichia canis* DNA was detected by conventional PCR using the primers gro-E.canis163s (5′-AAA TGT AGT TGT AAC GGG TGA ACA G-3′) and gro-E.canis573as (5′- AGA TAA TAC CTC ACG CTT CAT AGA CA-3′), as described elsewhere [25]. Each reaction contained 7.5 μl of DNA-free water, 1.5 μl of each primer at a concentration of 10 pmol/μl, 12.5 μl GoTaq^®^ Colorless Master Mix (Promega, Madison, USA) and 2 μl of the sample DNA to be tested. Thermal cycling conditions were as follows: denaturation at 95 °C for 30 s, followed by 40 cycles of 94 °C for 10 s, 62 °C for 15 s and 72 °C for 15 s, with a final extension step at 72 °C for 1 min. DNA extracted from the blood of a dog infected by *E. canis* was used as positive control and a master mix with no DNA as NTC.

All conventional PCR assays were run on a Veriti^®^ 96-Well Thermal Cycler (Applied Biosystems, Foster City, CA, USA) and amplicons were analysed by 1.5% agarose gel electrophoresis and visualized in ultraviolet light.

### Statistical analysis

Baseline prevalence was calculated for each of the two study locations considering dogs positive to one or more tests. Baseline prevalence for *Leishmania* spp. was calculated using data from all dogs initially screened, whereas for other pathogens it was calculated using data from dogs included in the longitudinal study. Exact 95% confidence intervals (95% CI) were calculated for each baseline prevalence.

YCI was expressed as percentage and calculated considering the number of dogs positive at the interim and/or at the final follow-up, using the following formula: number of positive dogs/(number of negative dogs at baseline − number of dogs lost to follow up) × 100. For calculation purposes, “dogs lost to follow up” were those tested at baseline but removed from the study before the interim follow-up (i.e. not retested in the study). Likewise, we used the last observation carried forward (LOCF) method, so data from dogs tested at the interim follow-up, but removed before the final follow-up were included in the calculations. Finally, dogs positive at the interim follow-up and eventually negative at the final follow-up were considered as positive.

The differences in baseline prevalence and YCI for each pathogen between dogs from São Joaquim de Bicas and Goiana were tested using Chi-square test, with a *P* < 0.05 considered statistically significant. Calculations and statistical analyses were performed using GraphPad QuickCalcs (http://www.graphpad.com/quickcalcs/) and BioEstat, version 5.3 (Instituto de Desenvolvimento Sustentável Mamirauá, Belém, Pará, Brazil).

## Results and discussion

Out of 823 dogs initially screened, 131 (15.9%; 95% CI: 13.6–18.6%) were positive for anti-*Leishmania* spp. antibodies or to *Leishmania* kDNA (Additional file 1: Table S1), with a higher positivity in São Joaquim de Bicas (19.5%) as compared to Goiana (14.7%), although this difference was not statistically significant. Our previous study with a much smaller sample size also suggested a higher prevalence of *Leishmania* spp. infection in dogs from São Joaquim de Bicas as compared to Goiana [19]. A previous study conducted in Natal (Rio Grande do Norte state, north-eastern Brazil) reported that higher owner education was associated with decreased levels of dog seropositivity to *Leishmania* spp. [26]. While the illiteracy rate is generally higher in Goiana as compared to São Joaquim de Bicas, a detailed analysis of individual dog owner education level would be necessary to assess whether this could be a main driver for the higher prevalence and YCI in São Joaquim de Bicas.

Out of the 307 privately-owned dogs enrolled in the longitudinal study, 120 (39.1%) were lost during the 1-year observation period for different reasons (e.g. animal death, owner decision, and lost to follow-up). The losses were higher in Goiana (*n* = 87; 42.7%) as compared to São Joaquim de Bicas (*n* = 33; 32.0%).

Comparative analyses revealed statistically significant differences between the baseline prevalence (Table 1) and/or YCI (Table 2) of some VBP infections in dogs from São Joaquim de Bicas and Goiana. In particular, the baseline prevalence and the YCI of *Anaplasma* spp., *D. immitis* and *Ehrlichia* spp. were significantly higher in Goiana. In contrast, the YCI of *Leishmania* spp. was significantly higher in São Joaquim de Bicas. The significantly higher YCI of *Leishmania* spp. infection in dogs from São Joaquim de Bicas suggests that dogs and people living in this municipality are at a higher risk of *Leishmania* spp. infection as compared to Goiana. According to official data at state level, the incidence of human visceral leishmaniasis in 2016 was 2.32 and 0.77 per 100,000 inhabitants in Minas Gerais and Pernambuco, respectively [15]. The higher incidence of human visceral leishmaniasis in Minas Gerais [15] may be due to many determinant factors, including higher exposure to sand fly vectors, which may be ultimately related to poor housing conditions, higher vector densities, or both.

**Table 1:** Baseline prevalence of various vector-borne pathogens in Goiana (Pernambuco) and São Joaquim de Bicas (Minas Gerais), Brazil

Pathogen^a^	Goiana		São Joaquim de Bicas		Statistics
	Prevalence (%)	95% CI	Prevalence (%)	95% CI	
*Anaplasma* spp.	45.1 (*n* = 204)	38.1–52.2	16.5 (*n* = 103)	9.9–25.1	*χ*^2^ = 24.44, *df* = 1, *P* < 0.0001
*Babesia* spp.	79.9 (*n* = 204)	73.7–85.2	81.6 (*n* = 103)	72.7–88.5	*χ*^2^ = 0.12, *df* = 1, *P* = 0.7304
*Dirofilaria immitis*	36.3 (*n* = 204)	29.7–43.3	0 (*n* = 103)	nc	*χ*^2^ = 49.23, *df* = 1, *P* < 0.0001
*Ehrlichia* spp.	64.7 (*n* = 204)	57.9–70.9	37.9 (*n* = 103)	28.5–48.0	*χ*^2^ = 19.99, *df* = 1, *P* < 0.0001
*Leishmania* spp.	14.7 (*n* = 516)	11.8–18.1	19.5 (*n* = 307)	15.5–24.4	*χ*^2^ = 3.24, *df* = 1, *P* = 0.0721

^a^Except for *Dirofilaria immitis* and *Borrelia burgdorferi* (not shown in the table), all pathogens were assessed by both serology and PCR. For more details, see “Methods” and Additional file 1: Table S1

*Abbreviations*: CI, confidence interval; *n*, number of dogs considered in the calculations; nc, not calculated

**Table 2:** Year-crude incidence of various vector-borne pathogens in Goiana (Pernambuco) and São Joaquim de Bicas (Minas Gerais), Brazil

Pathogens^a^	Goiana		São Joaquim de Bicas		Statistics
	Year-crude incidence (%)	95% CI	Year-crude incidence (%)	95% CI	
*Anaplasma* spp.	38.3 (*n* = 112)	27.7–49.7	7.1 (*n* = 75)	2.4–15.9	*χ*^2^ = 12.76, *df* = 1, *P* = 0.0004
*Babesia* spp.	96.0 (*n* = 49)	79.7–99.9	100.0 (*n* = 26)	75.3–100.0	*χ*^2^ = 0.01, *df* = 1, *P* = 0.9330
*Dirofilaria immitis*	39.8 (*n* = 130)	30.4–50.0	1.3 (*n* = 81)	< 0.1–6.8	*χ*^2^ = 25.05, *df* = 1, *P* < 0.0001
*Ehrlichia* spp.	58.5 (*n* = 84)	44.1–71.9	22.9 (*n* = 59)	12.0–37.3	*χ*^2^ = 5.57, *df* = 1, *P* = 0.0183
*Leishmania* spp.	19.6 (*n* = 177)	13.5–26.9	43.8 (*n* = 115)	32.7–55.3	*χ*^2^ = 8.04, *df* = 1, *P* = 0.0046

^a^Except for *Dirofilaria immitis* and *Borrelia burgdorferi* (not shown in the table), all pathogens were assessed by both serology and PCR. For more details, see “Methods”

*Abbreviations*: CI, confidence interval; *n*, number of dogs considered in the calculations; nc, not calculated

As for the other VBPs, higher prevalence values for *Anaplasma* spp., *D. immitis* and *Ehrlichia* spp. were found in Goiana, as compared to São Joaquim de Bicas, which agrees with our previous observations [19]. This is probably due to a higher pressure of ectoparasites in Goiana, which may be related to the infrequent use of ectoparasiticides on dogs, as discussed elsewhere [19]. Indeed, the socioeconomic profile of inhabitants living in the studied municipalities differs in terms of illiteracy rates, human development index, and gross domestic product per capita, according to official data from the Brazilian Institute of Geography and Statistics [27]. In addition, the dog owner attitude towards ectoparasite control also differs, in which the frequency of ectoparasite treatments was found to be higher in São Joaquim de Bicas as compared to Goiana in a previous study [19]. Thus, one could speculate that the higher prevalence of infection by *Anaplasma* spp. and *Ehrlichia* spp. in Goiana could be also related to a lower frequency of ectoparasite treatments in dogs from this municipality. However, *Babesia* spp. infection, contracted *via* tick infestation, occurred in high prevalence in both regions (further discussed below). The above hypothesis also does not apply to *D. immitis*, considering that this parasite does not seem to be endemic in São Joaquim de Bicas [19], as in other municipalities of Minas Gerais [16]. On the other hand, *D. immitis* is highly prevalent in Goiana, as in other municipalities of Pernambuco [16, 19, 28]. The finding of a single faintly positive dog in São Joaquim de Bicas at the follow-up may be a result of a rare, but possible cross-reactivity reaction with other parasite antigens [29].

While the serological tests used herein are not always species-specific to *A. platys*, *E. canis* and *B. vogeli*, these are by far the most widespread species belonging to these genera in Brazil [7], including in Pernambuco [30]. For instance, *A. phagocytophilum* has been reported in some occasions in south-eastern Brazil [31], but so far not in the north-eastern region of the country. *Ehrlichia ewingii* has been suspected in five dogs in south-eastern Brazil [32], but this finding needs further confirmation as reviewed elsewhere [33]. Finally, *Babesia gibsoni* has been reported on rare occasions in dogs from the southernmost part of Brazil [34], but not elsewhere in this country.

The YCI of some VBPs herein calculated was generally similar to that recorded by molecular and serological tests in sheltered young dogs in southern Italy [25, 35, 36]. In spite of the higher prevalence and YCI of *Anaplasma* spp. and *Ehrlichia* spp. in Pernambuco, no significant difference was found in relation to *Babesia* spp. This is also in line with our previous study, in which we reported high seropositivities for *Babesia* spp. in both Goiana and São Joaquim de Bicas [19]. This is expected considering that dogs living in the study areas were frequently seen infested by ectoparasites, including *Rhipicephalus sanguineus* (*sensu lato*) ticks and *Ctenocephalides* spp. fleas (data not shown), as reported previously [19].

Because both *E. canis* and *B. vogeli* are transmitted by *R. sanguineus* (*s.l.*), there is no plausible explanation for the similar prevalence of *Babesia* spp. and the dissimilar prevalence of *Ehrlichia* spp. in Goiana and São Joaquim de Bicas, but maybe this could be related to distinct patterns of antibody production in dogs against these agents. Considering that the vector competence of different populations of *R. sanguineus* (*s.l.*) for *E. canis* has already been documented [37], our results may suggest that the tick population present in Goiana may be more capable of transmitting *E. canis* as compared to the population of São Joaquim de Bicas. Nonetheless, this could also be related to seasonal patterns to tick infestations on dogs in these municipalities, which could somehow be affecting *E. canis*, but not *B. vogeli* transmission.

Anti-*Borrelia burgdorferi* antibodies were not detected in any of the samples from Goiana and São Joaquim de Bicas, which was expected considering the absence of competent vectors in the study areas. While some rare positive results have been reported in studies conducted in Brazil and other Latin American countries [38], further studies are needed to confirm the presence and significance (if any) of borreliosis in dogs from this part of the world.

Longitudinal studies on the very same population of dogs are challenging to conduct, especially with privately-owned dogs that are untreated against ectoparasites. One of the major challenges is the high proportion of dogs lost to follow-up, as reported in this study. Therefore, data generated herein will be useful for future studies and analyses, perhaps also for different pathogens not considered herein.

## Conclusions

This study confirms high prevalence and YCI of various VBPs in dogs in two geographical regions of Brazil. Our data also indicate that the risk of infection varies significantly for individual pathogens and between the regions, which may be related to several biotic (e.g. climate and socioeconomic status of dog owners) and abiotic (e.g. vector competence and capacity of different tick populations) factors that are still poorly understood. YCI may be somewhat underestimated in this study considering the ectoparasitic treatments that some dogs received and considering that the LOCF method for missing data at the final follow-up was applied to all dogs concerned, including dogs that were negative at the interim. Such dogs might have become positive until the final follow-up, but were considered to be negative within the analyses. Still, the impact is expected to be low as only limited numbers of dogs were concerned.

## Supplementary information

**Additional file 1: Table S1.** Total number of dogs tested by serology and PCR for different pathogens at each time point (baseline, after 8 and 12 months), in Goiana (Pernambuco) and São Joaquim de Bicas (Minas Gerais), Brazil. Numbers are expressed as “positive/total (positivity)”.

## Data Availability

The data supporting the conclusions of this article are included within the article and its additional files. Raw data can be shared with other researchers upon specific request.

## Abbreviations

CI: Confidence interval
CNPq: Conselho Nacional de Desenvolvimento Científico e Tecnológico
ELISA: Enzyme-linked immunosorbent assay
IFA: Indirect immunofluorescence
LOCF: Last observation carried forward
NTC: No template control
PCR: Polymerase chain reaction
VBP: Vector-borne pathogens
YCI: Year-crude incidence
