# Supplementary material for: Prevalence and incidence of vector-borne pathogens in unprotected dogs in two Brazilian regions
Source: Parasit Vectors. 2020 Apr 21;13:195. doi: 10.1186/s13071-020-04056-8 (PMC7171771; doi:10.1186/s13071-020-04056-8)
Supplement: Supplementary file 1 — Additional file 1: Table S1. Total number of dogs tested by serology and PCR for different pathogens at each time point (baseline, after 8 and 12 months), in Goiana (Pernambuco) and São Joaquim de Bicas (Minas Gerais), Brazil. Numbers are expressed as “positive/total (positivity)”. [file 13071_2020_4056_MOESM1_ESM.doc]

**Additional file 1: Table S1**. Total number of dogs tested by serology and PCR for different pathogens at each time point (baseline, after 8 and 12 months), in Goiana (Pernambuco) and São Joaquim de Bicas (Minas Gerais), Brazil. Numbers are expressed as “positive/total (positivity)”

| Pathogens | Test | Goiana | | | São Joaquim de Bicas | | |
| --- | --- | --- | --- | --- | --- | --- | --- |
|  |  | Baseline | 8 months | 12 months | Baseline | 8 months | 12 months |
| *Anaplasma* spp. | Serology | 82/204 (40.2%) | 53/148 (35.8%) | 47/117 (40.2%) | 15/103 (14.6%) | 8/80 (10.0%) | 7/70 (10.0%) |
|  | PCR | 17/204 (8.3%) | 15/148 (10.1%) | 12/117 (10.3%) | 3/103 (2.9%) | 1/80 (1.2%) | 0/70 (0.0%) |
| *Babesia* spp. | Serology | 158/204 (77.4%) | 139/148 (93.9%) | 116/117 (99.1%) | 83/103 (80.6%) | 62/80 (77.5%) | 69/70 (98.6%) |
|  | PCR | 14/204 (6.9%) | 4/148 (2.7%) | 5/117 (4.3%) | 7/103 (6.8%) | 1/80 (1.2%) | 4/70 (5.7%) |
| *Borrelia burgdorferi* | Serology | 0/204 (0.0%) | 0/148 (0.0%) | 0/117 (0.0%) | 0/103 (0.0%) | 0/80 (0.0%) | 0/70 (0.0%) |
| *Dirofilaria immitis* | Serology | 74/204 (36.3%) | 78/148 (52.7%) | 73/117 (62.4%) | 0/103 (0.0%) | 0/80 (0%) | 1/70 (1.4%) |
| *Ehrlichia* spp. | Serology | 129/204 (63.2%) | 118/148 (79.7%) | 92/117 (78.6%) | 39/103 (37.9%) | 37/80 (46.2%) | 37/70 (52.9%) |
|  | PCR | 21/204 (10.3%) | 38/148 (25.7%) | 12/117 (10.3%) | 5/103 (4.8%) | 12/80 (15.0%) | 2/70 (2.9%) |
| *Leishmania* spp. | Serology | 65/516 (12.6%) | 3/148 (2.0%) | 5/117 (4.3%) | 60/307 (19.5%) | 10/80 (12.5%) | 9/70 (12.9%) |
|  | PCR | 6/417 (1.4%) | 16/148 (10.8%) | 6/117 (5.1%) | 1/215 (0.5%) | 22/80 (27.5%) | 14/70 (20.0%) |
